# Widening the Gates: Redefining Excellence in Selection for Health Professions Education for a Diverse Future Workforce

**DOI:** 10.5334/pme.1295

**Published:** 2024-08-30

**Authors:** Suzanne Fikrat-Wevers, Karen M. Stegers-Jager, Walter W. Van Den Broek, Andrea M. Woltman

**Affiliations:** 1 Institute of Medical Education Research Rotterdam, Erasmus MC University Medical Center Rotterdam, Rotterdam, The Netherlands; 2 Radboudumc Health Academy, Radboudumc, Nijmegen, The Netherlands

## Abstract

To ensure diversity in the healthcare workforce selection committees must select a cohort of students who collectively possess the wide variety of qualities necessary to serve societal needs. In practice, selection procedures primarily focus on predicting academic outcomes, which are currently based on a limited set of qualities, restricting the definition of excellence in healthcare. The authors propose a shift in the design of selection procedures by including additional considerations – student diversity and applicant perception – to select talented students who can fulfil societal needs. The authors explain the importance of incorporating these considerations into the design of selection procedures and challenges that may arise. To overcome the challenges of incorporating student diversity and applicant perception in the design of selection procedures, a new view on alignment between the profession, training and selection is needed. This starts with redefining excellence in the profession with more explicit attention to equity, diversity and inclusion (EDI). The authors argue that by employing an EDI-adjusted model of alignment, selection procedures can enhance academic outcomes, properly recognize the talents of and acknowledge the needs for a diverse future workforce and be perceived as fair by applicants.

Healthcare professionals need a wide variety of competencies to function optimally in today’s society. Many factors are influencing the performance and behaviour of healthcare professionals, including individual and organizational factors. Selection committees, in their role as gatekeepers to health professions education, have the power to decide on the individual factors that determine who ultimately will become health professionals to provide high-quality care to a diverse patient population. Given this responsibility, they should aim to select a cohort of students who, as a group, possess the wide variety of qualities necessary to serve societal needs. This includes emerging concepts such as equity, diversity, inclusion and social justice, physician humanism, data-informed medicine, and planetary health [1,2]. In practice, selection procedures are often designed with a focus on improving academic performance [3,4]. However, academic performance is currently based on a limited set of skills, narrowing the definition of excellence by overlooking other vital attributes that can contribute to an excellent healthcare workforce. Academic performance is currently based on what medical schools *can* easily measure in assessments during training, which does not adequately reflect the competencies that medical schools consider important and *would like to* or *should* measure. For instance, medical programs often struggle to teach and assess communication skills and intercultural competencies [5,6]. This is further complicated by the fact that there can be different perspectives on what constitutes proficiency in a skill, depending on cultural background, for example [7,8]. Consequently, concerns exist that the abilities among students from minority backgrounds are particularly devalued, evident in the “leaky pipeline” phenomenon. This phenomenon indicates the gradual loss of diversity in the path from undergraduate student to professional [9]. Consequently, health professions schools run the risk of missing students who could enhance the quality of healthcare, but whose talents lie beyond this limited idea of excellence.

This paper proposes a shift in the design of selection procedures in which institutions take the responsibility to select those cohorts of students who can collectively fulfil societal needs. We advocate for including additional considerations beyond academic performance, particularly student diversity and applicant perceptions. We delve into the importance of incorporating these factors and explore the challenges they present by addressing how to balance performance and student diversity, performance and applicant perceptions and student diversity and applicant perceptions. Finally, we propose an adjusted model of alignment to overcome these challenges. This model allows programs to prioritize predicting academic performance, whilst properly recognizing the talents of and acknowledging the needs for a diverse future workforce while ensuring fairness in the eyes of applicants. While we acknowledge the value of holistic and contextualized admissions strategies in diversifying student cohorts within medical education, such approaches are not always feasible due to legal constraints or institutional limitations. Therefore, this paper specifically addresses scenarios where holistic or contextualized admissions cannot be implemented.

## Why Should We Worry About Student Diversity and Applicant Perceptions?

There are two key ways in which current selection procedures are prone to missing talented students who could contribute to the future healthcare workforce. The first issue pertains to student diversity. A diverse healthcare workforce is, aside from issues of equity and fairness, important to increase and equalize access to high quality healthcare for the whole population [10,11]. For instance, healthcare providers from diverse backgrounds can better understand and address the unique needs and concerns of patients from different communities [12,13]. Furthermore, a diverse healthcare workforce can contribute to the quality of education and research [11]. For example, diversity can facilitate a research agenda that includes health problems that primarily affect the health of underrepresented groups [12]. However, despite the significance of a diverse healthcare workforce, selection procedures often yield negative outcomes for student diversity, as applicants from lower socioeconomic and ethnic minority backgrounds often have lower admission chances [14,15,16,17,18]. Consequently, broadened or non-cognitive criteria were introduced to address disparities in selection outcomes, but they do not consistently achieve the desired goal. Although research has shown that broadened criteria can reduce socioeconomic disparities, their impact on ethnic disparities is inconsistent and potentially context-dependent [15,16,17].

A second issue that can hinder the selection of a wide variety of talented students required to serve societal needs, albeit receiving less attention in the field of health professions education, relates to applicant perceptions. Applicant perceptions of selection are commonly described using organizational justice theory, which refers to the perception of fairness in the outcome of selection as well as in the procedure and methods that are used to generate this outcome [19]. Applicant perceptions of fairness can have practical impact on applicants at multiple stages. First, perceptions of the selection procedure could impact the decision whether to apply for a health professions program [20,21]. As an example, first-generation university students are less likely to choose medical school based on the curriculum than students whose parent(s) attended higher education and non-Western students are less likely to indicate the curriculum as the main reason for medical school choice than are Dutch students [21]. Additionally, negative perceptions can have negative consequences for applicant motivation and performance and can even cause withdrawal once applicants are undergoing the selection procedure [22,23,24,25]. Notably, this may impact the ability of selection to admit a diverse student cohort, since applicants from lower socioeconomic and ethnic minority backgrounds more often demonstrate negative perceptions of selection, which is often due to experiences of unequal access to resources [20,26,27] For instance, talented students from underrepresented backgrounds may underestimate their selection chances or feel disconnected due to a perceived misalignment with the medical program [26,28]. The image of the program that is communicated through the selection procedure can also impact its ability to identify talents in other ways. For instance, a lack of alignment between the collaborative skills needed in the future profession and a selection procedure which focuses on individual qualities inherently promoting competition, can result in a mismatch between the qualities of selected students and what society needs [29]. Strikingly, a highly demanding selection procedure requiring much preparation can negatively impact applicant well-being and can implicitly promote a culture of competitiveness amongst applicants [30,31].

## Balancing Multiple Factors in Selection

In selecting a student body that will meet societal needs, selection procedures should balance those societal needs with those of the applicants and schools. However, in practice, balancing these diverse needs can be challenging, as conflicts may arise between different interests. In the next sections, we delve into the conflicts and commonalities between performance, diversity, and applicant perceptions.

### Balancing performance and student diversity

A first challenge lies in balancing performance and student diversity. As previously mentioned, selection procedures are typically designed primarily to predict future academic performance, such as grades and completion rates. It is evident that enhancing academic performance is desirable, based on the expectation that it predicts future job performance [32]. Nevertheless, the goal of predicting academic performance is also, at least partly, encouraged by national policy: financial incentives are often provided based on completion rates [33]. Initiatives to combat unequal opportunities usually focus on either adjusting the selection procedure by introducing new selection tools [20] or by equalizing access to resources to prepare for an existing selection procedure [34,35,36]. We will reflect on both types of initiatives, and how these relate to the ability of selection procedures to predict future performance.

The introduction of alternative tools is often viewed as a challenge regarding balancing diversity and predicting academic performance, which is called the diversity-validity dilemma. Originating from personnel psychology, this concept suggests that selection tools with higher predictive validity also tend to have higher adverse impact on diversity, and the other way around [37]. This dilemma has resulted in efforts to increase student diversity by searching for selection tools that still have high predictive validity, while limiting adverse impact [15,16]. In other words, the focus is on how to *reduce* the harm on student diversity, rather than completely *eliminating* adverse impact or potentially even *promoting* student diversity. Moreover, the premise of this dilemma is that diversity and validity cannot go together in selection. In our opinion, the diversity-validity dilemma has multiple challenges, at least in the field of health professions education. Firstly, this dilemma arguably overlooks the assumption that subgroups do not fundamentally differ in their ability to, in this case, successfully complete an educational program. Although certain individuals probably possess greater aptitude for a future career in health professions, we firmly believe that such suitability is not related to one’s demographic characteristics. This is especially true in the case of selection for undergraduate higher education, where applicant pools are often already preselected based on academic skills due to stratified secondary education [38,39]. Consequently, these pools tend to be relatively homogeneous in terms of educational background, and in their potential to successfully complete health professions education, regardless of their sociodemographic background [17,18]. A second weakness of the diversity-validity dilemma pertains to the measurement of predictive validity. Since the validity of selection tools is usually assessed using academic performance, tools that promote student diversity may appear less valid than they actually are for two reasons. First, a lack of inclusion during the training and/or biased assessment can hamper the academic performance of students from underrepresented backgrounds [40]. Second, when valuable skills that applicants from underrepresented backgrounds bring to the profession are not adequately valued during training and assessment, this can result in a lack of association between selection outcomes and academic performance.

A second approach institutions use to enhance student diversity is by intervening in the preparation phase. To combat unequal access to commercial coaching [41,42,43,44], some institutions provide free preparatory activities – including summer schools or coaching days – to all or subgroups of applicants with the aim to equalize the starting points for applicants from different backgrounds in the selection procedure [34,42,44]. Although research thus far has shown mixed results regarding whether such activities are effective in increasing admission outcomes [42,44,45], the provision of such activities may have a modest contribution to student diversity. In our recent study, participation in such activities appeared to be particularly helpful in increasing the selection outcomes of some underrepresented subgroups of applicants [44]. Nevertheless, this positive association was not maintained once students were enrolled in the medical program, which may again be caused by a lack of inclusion and biased assessment in the program. Previous work revealed underperformance for underrepresented student groups especially in clinical training [46]. The limited success of preparatory activities is likely attributable to their reproduction of a limited definition of excellence by the institution, which is insufficiently aligned with societal needs and adopts a deficit approach without actually valuing the talents of applicants from non-traditional backgrounds [47]. However, it could also suggest the existence of a coaching effect [42,48] introducing a possible conflict between increased diversity via preparatory activities and predictive validity of selection.

### Balancing performance and applicant perceptions

Like balancing predictive validity and diversity, it can be challenging to reconcile predictive validity with applicant perceptions. Some tools that are currently considered to be highly predictive receive low levels of acceptance from applicants and the other way around. For instance, while applicants hold negative perceptions towards the use of pre-university grades as a selection method into undergraduate programs [30], pre-university grades are considered one of the strongest predictors of future academic performance [46]. Notably, in the case of other tools, acceptability and predictive validity are both high, such as in the case of curriculum-sampling tests [30,49]. Moreover, in a recent qualitative study we found that applicants underscore that selection should be aligned with the curriculum, in order to find the best ‘fit’ [31], and applicants are generally opposed to the use of lotteries that have recently been reintroduced in the Netherlands [25,30]. A selection procedure that is aligned with the curriculum, assessment, and indicators of future job performance has been shown to be highly predictive of academic outcomes [50]. Nevertheless, other values expressed by applicants may compete with predictive validity. For example, applicants express a strong desire for selection committees to communicate transparently about the selection criteria [31], which introduces another potential source of friction. Although transparent communication equalizes access to information regarding the procedure, it can result in applicants ‘gaming the system’ by shaping their behaviour towards what they think is expected from them [51,52]. Examples of such behaviour are “telling them what they want to hear and do whatever you have to do to get in”.

### Balancing student diversity and applicant perceptions

On the surface, applicant perception of selection can also be at odds with promoting student diversity. For example, the downside of the aforementioned high acceptability of curriculum-sampling tests is that they may be susceptible to ethnic performance disparities [17]. Applicants prefer to have a sense of control over their selection outcomes and demonstrate a desire to express who they are during the selection procedure [30]. This can be related to the cultural context: societies in the global north have been adopting meritocratic values, implying that success and failure can be attributed to one’s own efforts and talents, and that luck and background do not play a role in this matter [29,53]. Nevertheless, our qualitative data regarding applicant perceptions suggest that applicants recognize that opportunities to prepare for and perform on selection tools are unequally distributed [30]. Applicants deem this undesirable as equitable admissions are hindered. In fact, they consider that selection should aim to generate a diverse student population to better serve societal needs [31].

## How Can We Meet Different Needs?

As has become clear, it can be challenging to meet different needs. Interestingly, while applicants believe that identifying students who match best with the curriculum and future profession and generating diversity can go hand-in-hand [31], from the perspective of institutions it appears that compromising is inevitable. Is the institutions’ perspective on which talents are deemed deserving of becoming future health professionals currently too narrow?

**Figure 1:**
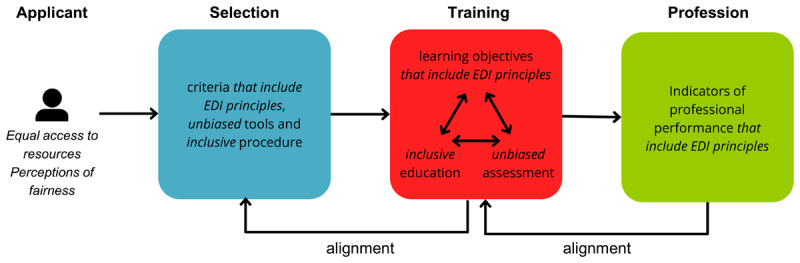
The EDI-alignment model. *Note*. EDI = equity, diversity and inclusion, cursive indicates additions to the model of alignment by Steenman [56]. Diversity (D) is the presence of perceived differences, equity (E) refers to the practice of promoting impartiality, fairness and access through processes and procedures that ensure individuals have equal opportunities based on their needs regardless of the systemic disadvantages or barriers put in their way. Inclusion (I) ensures that everyone feels valued and welcomed, while an inclusive workplace ensures individuals are empowered and provided with opportunities to meaningfully and authentically contribute [57].

Previously, researchers have argued that to enhance student diversity, efforts should rely not only on selecting a diverse student population, but also on preventing the loss of diversity during the training. For instance, the Attraction-Selection-Inclusion-Retention and Getting in Getting on frameworks address the importance of an inclusive learning environment [34,54]. While we agree that this is essential for promoting student diversity, programs should go a step further to better value the skills of students from underrepresented backgrounds. This can be achieved by shedding new light on alignment, a concept briefly mentioned earlier. Alignment is desirable from both the applicant’s and institutional perspective. However, to achieve greater diversity, it is important not to isolate initiatives but to consider the entire educational continuum. Therefore, we propose an EDI-alignment model (Figure 1). This starts with expanding the definition of excellence in healthcare by improving competency frameworks for the profession. Critics argue that current frameworks such as the CanMEDS do not sufficiently incorporate competencies related to equity, diversity, and inclusion [2,55]. When competency frameworks better address EDI, they will also better reflect the attributes of a diverse workforce. Subsequently, these competencies should be integrated and valued within the curriculum. An inclusive learning environment and unbiased assessment should support diversity retention throughout the pipeline, whilst also enabling a more accurate measurement of the validity of a selection procedure. Finally, these competencies should be reflected in the selection procedure and measured in an unbiased manner. Only by seeing diversity along such a continuum can validity and diversity truly coexist.

The proposed approach – the EDI-alignment model – distinguishes itself from holistic admissions in several key aspects. Firstly, it encompasses the entire educational continuum by embedding diversity across all facets of education, not solely focusing on admissions. Secondly, while holistic admissions explicitly consider background characteristics, the adjusted model of alignment takes a more implicit route in acknowledging candidate backgrounds. In some regions where explicit consideration of such characteristics is prohibited, the EDI-alignment model values qualities often associated with underrepresented backgrounds without directly assessing them. Nevertheless, holistic admissions provide valuable insights that can inform the implementation of this approach.

The proposed EDI-alignment model will enable schools to meet the need for selection and assessment strategies that value academic performance while fostering student diversity and applicant perception, leading to a more diverse and highly qualified healthcare workforce. We acknowledge that the EDI-alignment model is a significant undertaking and requires long-term investments in both research and implementation. Many questions remain to be answered, such as: What competencies are needed to provide appropriate care in a growing diverse society? How can we translate these competencies into measurable constructs that can be assessed at the start of the educational program? How can we validly measure those skills? How can applicant well-being and their desire for transparency be taken into account when assessing these skills? These questions are not easy to answer, and their answers may require major revisions of the expected learning outcomes, educational programs and selection procedures. However, educational institutions are responsible for at least recognizing the magnitude of this issue and its call for institutional changes. Merely implementing individual interventions in selection procedures and/or the educational program is not sufficient to improve diversity. If educational institutions recognize this, it might be easier for them to temporarily set aside their focus on academic performance and invest in (research into) the necessary institutional change. In the long run, this will eventually enhance academic outcomes and, more importantly, the quality of healthcare.

Thus, if institutions truly acknowledge the significance of diversity, they must align their actions accordingly, just as applicants expect them to.
